# Efficiency of the immunome protein interaction network increases during evolution

**DOI:** 10.1186/1745-7580-4-4

**Published:** 2008-04-22

**Authors:** Csaba Ortutay, Mauno Vihinen

**Affiliations:** 1 Institute of Medical Technology, FI-33014 University of Tampere, Finland; 2 Tampere University Hospital, FI-33520 Tampere, Finland

## Abstract

**Background:**

Details of the mechanisms and selection pressures that shape the emergence and development of complex biological systems, such as the human immune system, are poorly understood. A recent definition of a reference set of proteins essential for the human immunome, combined with information about protein interaction networks for these proteins, facilitates evolutionary study of this biological machinery.

**Results:**

Here, we present a detailed study of the development of the immunome protein interaction network during eight evolutionary steps from Bilateria ancestors to human. New nodes show preferential attachment to high degree proteins. The efficiency of the immunome protein interaction network increases during the evolutionary steps, whereas the vulnerability of the network decreases.

**Conclusion:**

Our results shed light on selective forces acting on the emergence of biological networks. It is likely that the high efficiency and low vulnerability are intrinsic properties of many biological networks, which arise from the effects of evolutionary processes yet to be uncovered.

## Background

Evolutionary pressures shaping protein interaction networks are practically unexplored, although the importance of such studies has been recognized [1]. The earlier studies addressing this question analyzed the conservation of network motives [2-5], or the rate of link dynamics in interaction networks [6,7]. Although the importance of different protein properties to the evolution rates of proteins has been questioned [8-10], proteins interacting with multiple partners simultaneously have been shown to have a slower pace of evolution [11]. Thus, the properties of interaction networks can be thought to influence the evolution of organisms. More general knowledge about the effectors shaping protein interaction networks would give insights on several aspects of the formation of complex biological systems.

For detailed analysis of the evolution of a protein-protein interaction (PPI) network it is necessary to have knowledge about the emergence of all the proteins in the network and their interactions. Some investigations in this line have been presented [12,13]. The problem is that the steps which lead to the current situation cannot be directly addressed. To be able to follow the development in biological systems, information about biological evolution should be applied. One attempt in this direction was to use so-called isotemporal categories to express the appearance of a gene or protein in organisms [14].

DNA and protein sequences hold their past in their structure, which can be reconstructed with the standard tools of phylogenetics. Here we performed to our knowledge the first PPI network analysis utilizing phylogenetic information. One of the reasons for the lack of this kind of study is the problem of obtaining accurate and validated data for a system that covers the biological network, its gene and protein constituents and their evolutionary history and homologs. We have collected such data for the human immune system.

The human immune system, which is one of the most complex biological machineries, has been widely investigated at the molecular, cellular and organ level in its normal state and during disease. It is a very complex system built up from several different tissues, cell types, molecules and processes. The evolutionary history of the human immune system is widely studied (see e.g. [15]), but we still need new data to complete the picture.

The Essential Human Immunome is a reference collection of genes and proteins involved in human immunity which is distributed in the Immunome database [16,17]. Evolutionary data for all these proteins is collected in the ImmTree database [18]. Experimentally verified protein-protein interaction data for the immunome proteins was collected from the Human Protein Reference Database (HPRD) [19] and used to reconstruct the protein interaction network.

Recently, several studies have been published about natural networks [20], ranging from social interactions [21,22] via protein-protein interactions [23,24] to the spreading of epidemics [25] as well as human made networks like telecommunication networks [26,27] and the Internet [28,29]. It was initially surprising that the different networks share several common characteristics, which suggests common organizing principles for their emergence [30].

Preferential attachment [31-33] suggests that a new protein is more likely to be connected to a highly connected protein than to a protein with fewer interactions. Networks built up according to this principle, have been shown to have scale free characteristics [34,35]. Scale free networks have been detected from several different sources, but thus far it has been very rare to observe the preferential attachment rule at work, especially in the case of protein interaction networks [36,37].

The evolution and development of networks has been widely studied [38,39]. Many earlier reports on the evolution of protein interaction networks have focused on yeast high throughput datasets [14,40,41]. Evolutionary comparisons have been limited to a few reference genomes. The new dataset on the human immunome [16] related proteins and their evolution allowed us to assign evolutionary levels to the proteins [18]. These levels can be interpreted as indicating the steps of emergence of proteins. Reliable protein interaction data can be assigned from HPRD, which contains experimentally verified interactions from literature [19]. We studied the emergence of the immunome PPI network and elucidated which of the network parameters are shaped by evolutionary pressures and how these parameters have changed during time.

## Results and discussion

The evolution of the immunome network was investigated by combining information about genes and proteins forming the immunome. 847 essential genes and proteins were identified by text and bioinformatic data mining as related to the human immune system [16]. The evolutionary history and relationships of the immunome proteins were obtained from ImmTree [18], which contains information on the orthologs for each protein. An evolutionary level was assigned for each protein. The level denotes when the protein emerged during evolution. The levels are presented in Table 1. The third essential component, the PPIs, came from the Human Protein Reference Database (HPRD) [19], which contains only experimentally proven interactions.

**Table 1:** Network parameters for the human immunome at the evolutionary levels of the corresponding subnetworks.

Evolutionary level	Taxon name	Nodes	Edges	α^a^	SE(α)^a^	-2logΛ^a^
0	*Homo sapiens*	584	1349	2.226	0.0677	1713
1	Mammalia ancestors	577	1335	2.235	0.0683	1699
2	Amniota ancestors	453	1039	2.260	0.0790	1304
3	Tetrapoda ancestors	357	770	2.332	0.0950	970
4	Vertebrata ancestors	354	761	2.319	0.0950	957
5	Chordata ancestors	159	254	2.607	0.1924	303
6	Coelomata	145	223	2.725	0.2144	267
7	Bilateria ancestors	106	124	3.478	0.4107	115
8	Fungi/Metazoa ancestors	62	57	4.376	0.9953	29
9	Eukaryota ancestors	48	39	4.585	1.5668	12

^a^Estimates for power law exponent (α) of the degree distributions are presented with the standard error [SE(α)] together and the log-likelihood ratios (-2logΛ) of the power law degree distributions.

Further, interactions for low level subnetworks, levels 6–9, were collected for *Drosophila melanogaster *[42,43] and *Caenorhabditis elegans *[42]. This was done to reconstruct early interactions between immunome proteins which might have been lost during the evolutionary processes. Only 13 new interactions were identified in these subnetworks and thus they did not affect the overall trends. It would have been beneficial to have PPI data for model organisms on all the 10 evolutionary levels. This is not currently possible due to lacking proteome wide PPI networks.

For the analysis, we made some assumptions. We used PPIs identified in the human proteome, and assumed that if two proteins which interact in human, they also interact in any organism in which the two proteins coexist. We also assumed that this interaction existed during the evolution since both of the proteins emerged in a linage. The model is a simplification of all possible cases. This assumption is also in use in interaction predictions [44,45], and although not exclusive, it is still true for the majority of interactions. Our results for interactions in fruitfly and worm confirm this idea, because hardly no new interactions were found in these low level subnetworks. The model simplifies the evolution of the entire interaction network to the evolution of its nodes. This is necessary since we do not have a method to track the evolutionary past of interactions, while the phylogenetic analysis of the proteins has well established and accepted procedures.

The human immunome PPI network contains 1349 interactions for 584 proteins (Table 1). Since the network includes only experimentally proven interactions from the HPRD database, we can assume it represents a real, albeit incomplete, network model of protein interactions in the human immune system. Not all the immunome proteins are included because the data is not complete and does not cover all proteins in all cell types and conditions.

Of the investigated proteins, protein-tyrosine kinase FYN has the highest number of interactions. The subnetwork of FYN and its first neighborhood includes 47 proteins and 93 interactions, which account for about 8% of all the immunome nodes and 6.9% of interactions. Another Src-family member, lymphocyte-specific protein tyrosine kinase (LCK), is the second most linked protein with 37 interactions. 64 of the 584 proteins have more than 10 interactions. Many of these are mediators of signal transduction pathways, for example the Janus kinases (JAKs), the signal transducer and activator of transcription (STAT) family members, and the TNF receptor-associated factors (TRAFs) (see Additional file 1).

**Figure 1:**
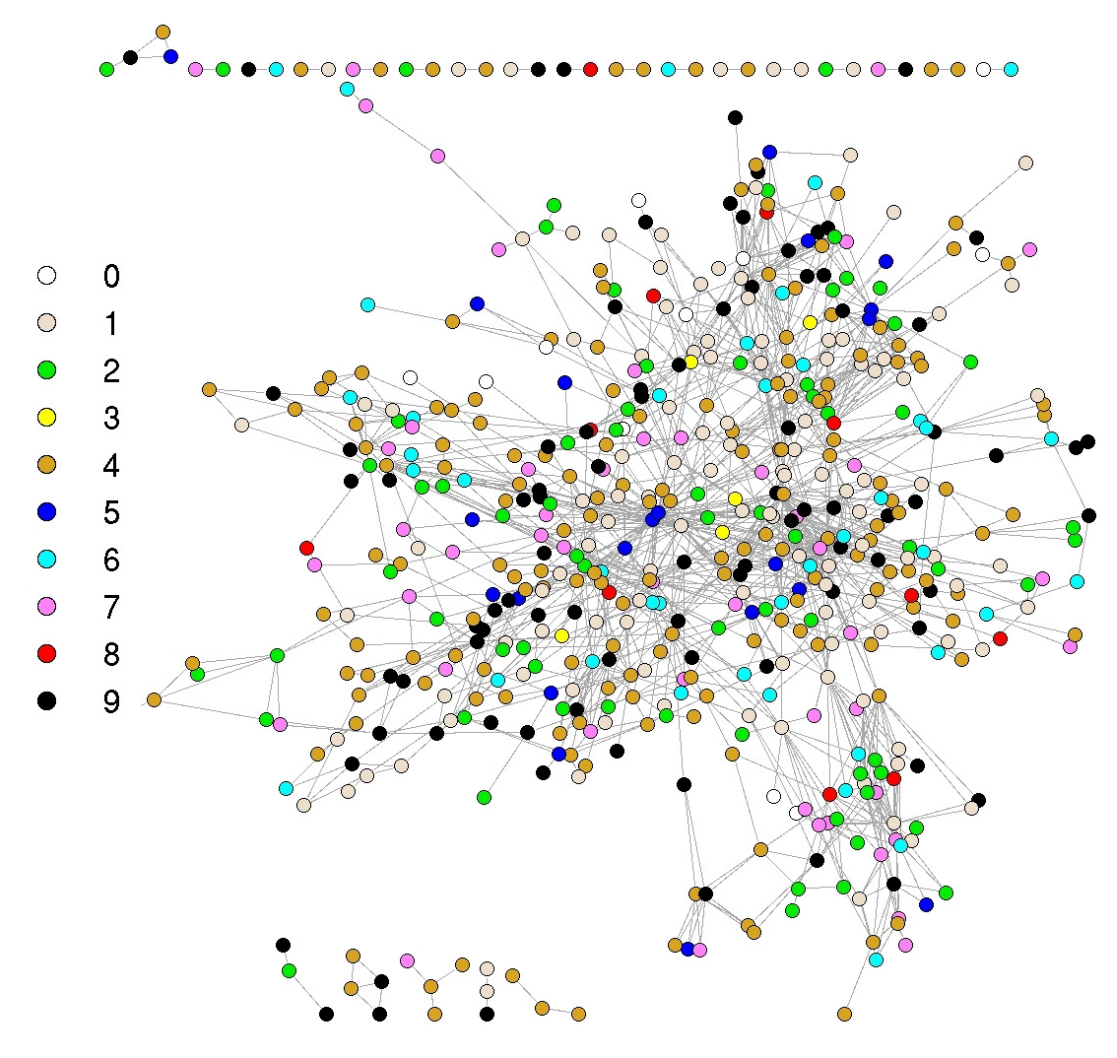
**Graph representation of the immunome network at the evolutionary levels.** Colors represent the levels of nodes as shown. See Additional file 2 for gene names and the network on the different evolutionary levels.

**Figure 2:**
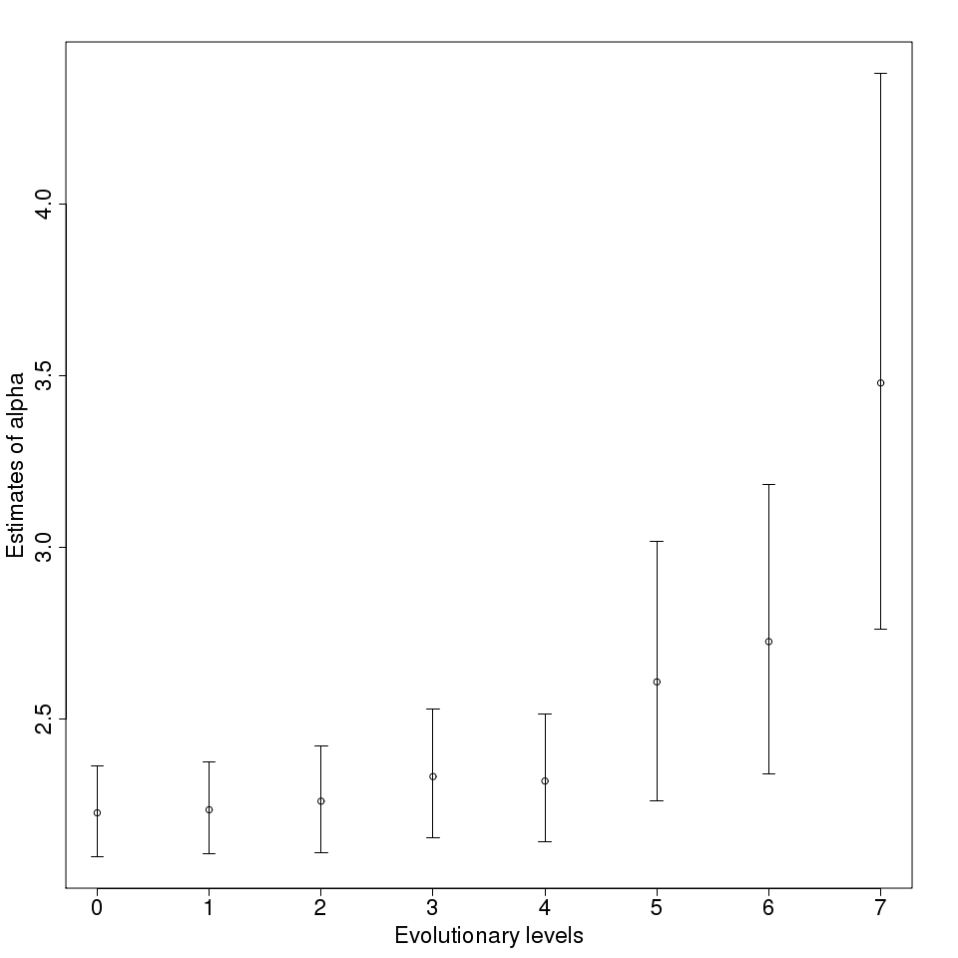
**Estimates for the power law exponents (α) of the networks on the different levels.** The bars show the confidence intervals with the 2.5% lower and 97.5% upper boundaries.

For further analysis, subnetworks were created for eight evolutionary levels. Levels 8 and 9 were excluded from the analysis because these levels contain only a few proteins and interactions. All subnetworks contain the nodes from the examined and earlier levels, and the interactions between them (Table 1, Fig 1, Additional file 2). They thus represent the interaction network that existed at different steps during evolution. The lowest, level 7, subnetwork is small, with 112 proteins and 133 interactions which existed in the ancestors of *Homo sapiens *when the taxon Bilateria was formed. In each evolutionary step the number of proteins and the interactions between them grows substantially. The degree distribution of the subnetworks follows power law distribution with the power law exponent between 2 and 3 (Fig 2.). The log-likelihood ratio (-2logΛ), which marks the likelihood of the power law degree distribution of the dataset, is much higher in the higher level networks (Table 1).

**Figure 3:**
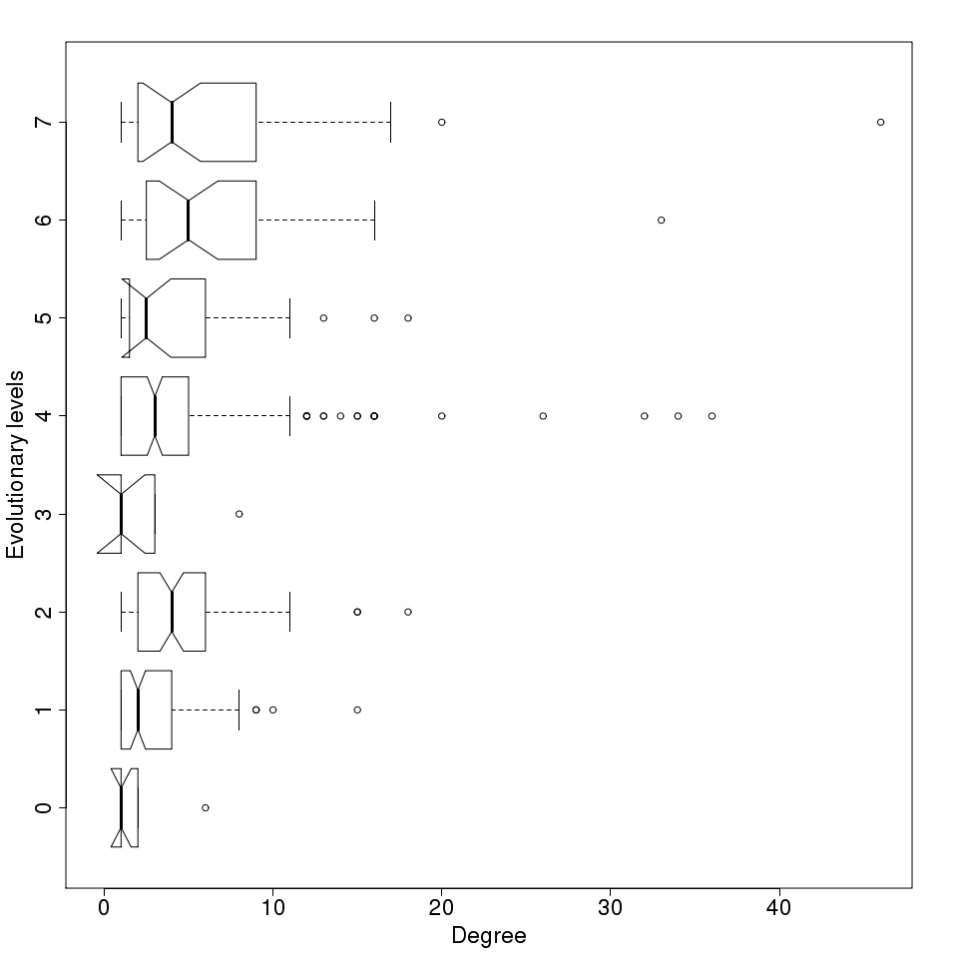
**Degree distribution for evolutionary levels of the protein-protein interaction network.** Notches represent the 95% confidence interval of the median.

**Figure 4:**
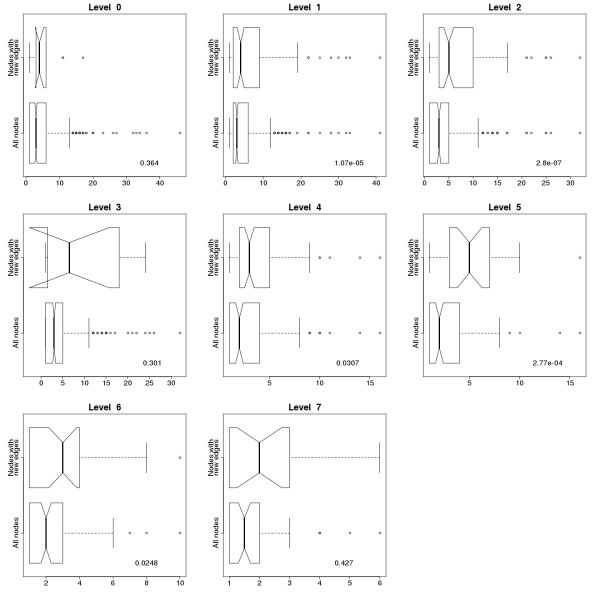
**Comparison of degree distributions of all the nodes and the nodes with new connections in the immunome protein interaction network at evolutionary levels 0–7.** Degree distribution for all the edges in the network is on the lower half of the subgraphs, while degree distribution only for nodes with new connections, representing the proteins with newly formed interactions, is on the upper half. P values for the Kruskal-Wallis Rank Sum Test are shown on the plots. Nodes with new connections have higher degrees than the others, and the difference is considered significant at levels 1, 2, 4, 5 and 6. Notches represent the 95% confidence interval of the median.

When analyzing the relationship between the evolutionary levels and the degrees (the number of interactions of the proteins) we expected proteins which appeared early on to have more interactions [37], and thus nodes with higher evolutionary levels should have higher degrees. However, a simple comparison of node level numbers and their degree in the level 0 network does not show this phenomenon (Fig 3). When we tested whether the new nodes introduced in evolutionary steps tend to attach to nodes with higher numbers of connections, a statistically significant preferential attachment is clear (Fig 4). We compared the degree distribution of all the nodes and of those nodes which get new connections in the next evolutionary level. The nodes with new connections have higher degrees in each step. This implies that when a new node is introduced into the immunome network, it most likely attaches to a node with a higher degree, so there is a bias toward attachment to higher degree nodes.

We used scale free models although the immunome protein interaction network, like other PPI networks, does not contain enough nodes to fulfill the statistical criteria for scale freeness. Therefore we mostly used general descriptive measures of networks, like efficiency, and avoided in our conclusions the scale free network specific aspects.

**Figure 5:**
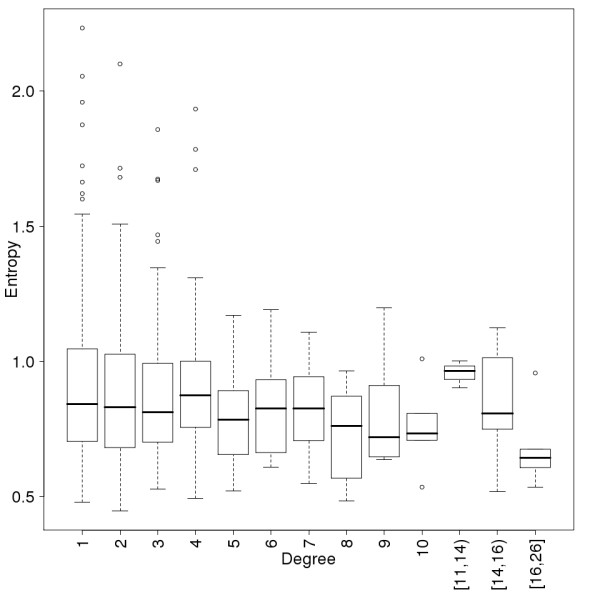
**Conservation of the proteins as a function of their connectivity in the human immunome PPI network.** Conservation is measured by the average entropy of the proteins. Data points are binned so that a minimum of 6 points are in each degree interval. The conclusion, that proteins with high connectivity never have high entropy, does not depend on the binning. The notation [11,14) means a bin for degree values 11, 12 and 13.

**Figure 6:**
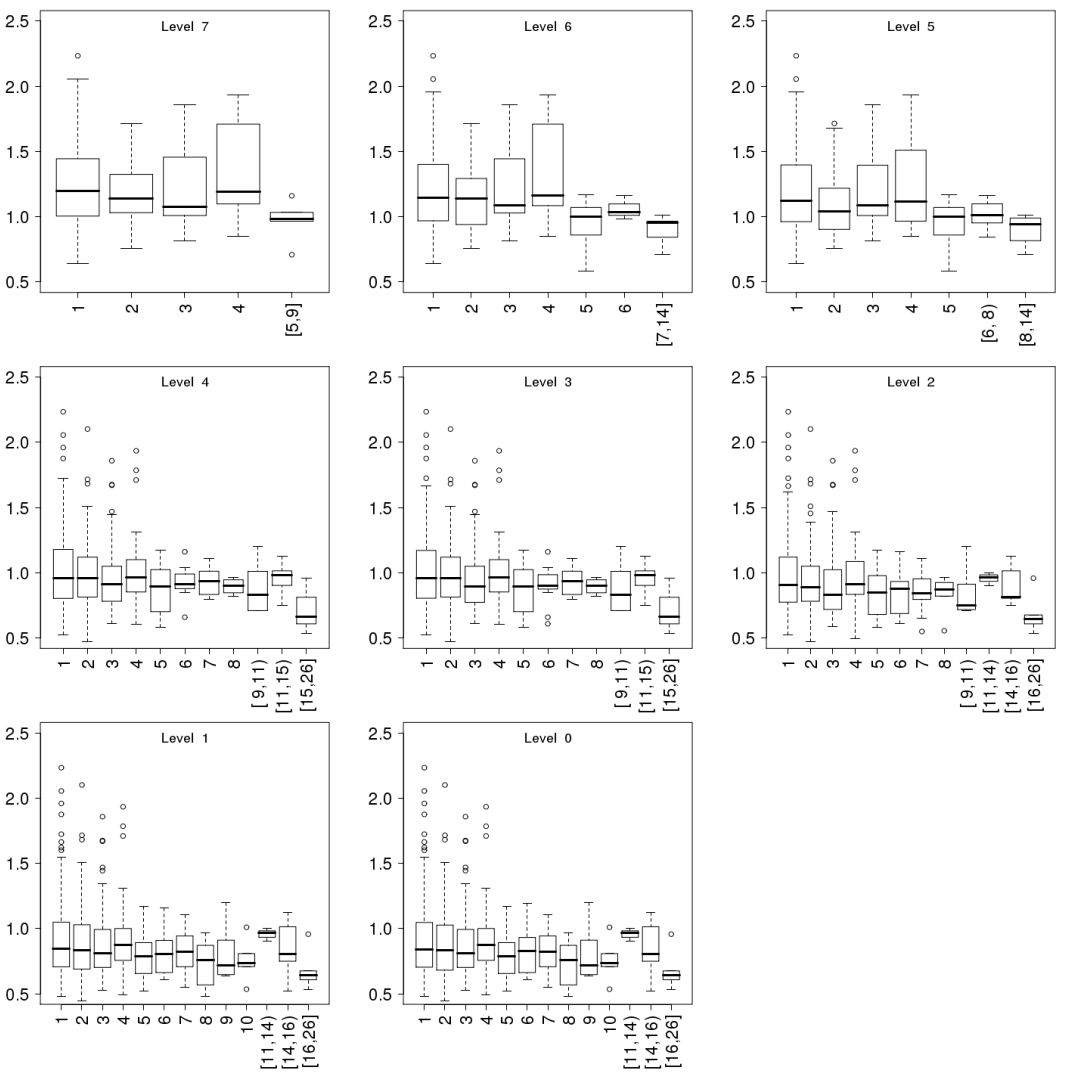
**Conservation of the proteins as a function of their connectivity in the immunome protein-interaction networks at the eight evolutionary levels.** Data is presented in the same way as for Fig 5.

An important feature of the scale free protein interaction networks is that highly connected nodes tend to be essential and therefore more conserved [40,46]. We used the average entropy [47] of the proteins to measure how conserved, and thus how essential they are. Entropy was used to measure the variability of the sites in a multiple protein sequence alignment instead of comparing a human sequence to an ortholog in a reference genome. We thus take variability into account from many sequences instead of a sequence pair. Proteins with high connectivity never have high entropy; yet on the other hand, some of the proteins with just a few connections have very high average entropy, which means that they are not conserved (Fig 5). We detect this phenomenon on all the evolutionary levels. These levels also contain enough nodes to allow binning of the data (Fig 6). More conserved proteins are more connected during the evolution of the immunome protein interaction network.

**Figure 7:**
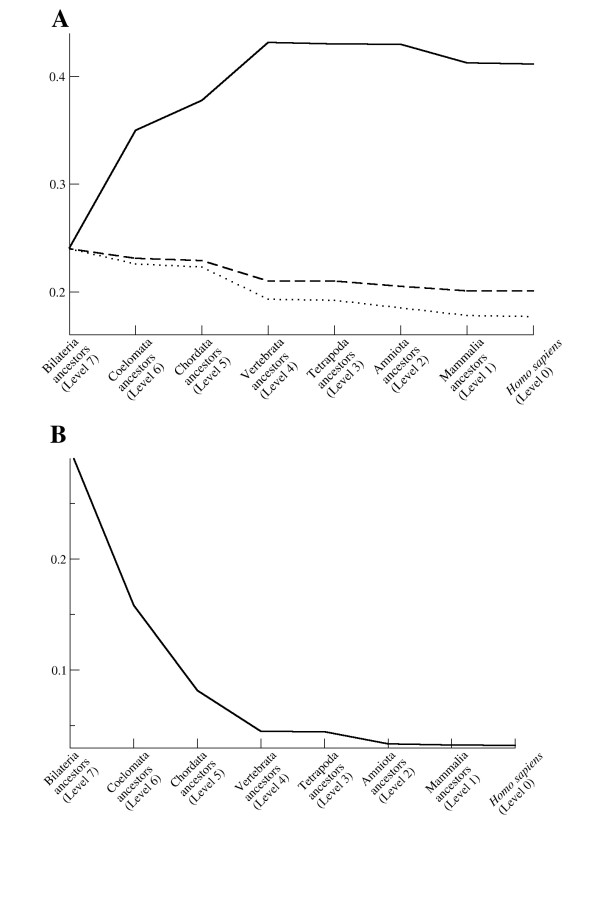
**Characteristics of the human immunome network during evolution.** (**A**) Efficiency (solid line) and expected efficiency calculated as a function of *1/(ln ln N) *(dashed line), and *1/(ln N) *(dotted line), where *N *is the number of nodes in the network. Expected efficiency curves are scaled to have the same starting values as the observed network. The shape of the observed efficiency curve shows an opposite trend than expected, suggesting that selective forces during evolution favor higher efficiency. (**B**) Maximal vulnerability.

We further studied the effects on several network characteristics during the evolution of the network to find out what kind of selective forces affect its development. Global efficiency quantifies the efficiency of the network in sending information between nodes [48]. According to earlier studies, the efficiency of a scale free network is expected to decrease when the size of the network is growing [36,48]. Surprisingly, the efficiency of the immunome network grows through the evolutionary steps, from an initial value of around 0.24 to a final value of around 0.41 although the number of nodes and edges is also growing (Fig 7A). This means that despite the number of nodes increasing from 112 to 584, the average number of steps necessary to reach one random node from another decreases. Since this is against the expected behavior (Fig 7A), we assume that a selection pressure exists which shapes the immunome PPI networks to became more efficient during evolution.

The critical components of a network can be searched by looking for the most vulnerable nodes [49]. Vulnerability is defined as the drop in efficiency when a node and all its edges are removed from the network. The maximal value of the vulnerability is the overall vulnerability of the whole network. The maximal vulnerability of the immunome network constantly decreases during the evolutionary steps from the initial value of 0.28 (Fig 7B). At the level of *Homo sapiens *(level 0) the value is 0.003, which means that maximal drop in the efficiency of the network is 0.3% if one of the nodes is deleted from the network. Scale free networks are known to be tolerant of errors in randomly chosen nodes [28], a feature that is also important for biological interaction networks.

## Conclusion

Our findings of the increasing efficiency and the decreasing vulnerability of the evolving network also raise new aspects in evolution. Apparently, it is advantageous for an interaction network to become more efficient and less vulnerable to random errors. More robust networks can possibly increase the fitness of the organism. It is likely that similar behavior will be found from some other biological, and possibly other types, of networks. Our analysis revealed new concepts for evolutionary biology, as well as new insights on the emergence of scale free networks. Large scale experimental studies are needed to test and verify the observed network properties and their effect during evolution.

## Methods

### Reconstructing the human immunome related protein-protein interaction network

Human immune system related proteins were collected from the Immunome database, which is a reference set for the human immune system composed by a combination of literature analysis and data mining [16]. Protein interactions were associated with the immunome proteins according to the Human Protein Reference Database (HPRD) [19]. Since only interactions between the immunome proteins were taken into account, no new nodes were added, but proteins without interactions were eliminated from the dataset. The final network contains 584 nodes out of the 847 original ones, forming altogether 1349 interactions. Interactions which appeared more than once were simplified to single edges.

### Generating subnetworks at ten levels of evolution

Evolutionary information was assigned to all the proteins of the network. The data was taken from the ImmTree database [18] which represents ten evolutionary emergence levels from Eukaryota (level 9) to *Homo sapiens *(level 0) (Table 1) [16]. The numbers are the unified evolutionary levels from the database. The emergence levels were defined by analysis of the orthologs of each protein, and they represent the earliest common ancestor of species where orthologs of a protein can be identified. For example in the case of the *FYN *gene for protein-tyrosine kinase fyn, the ortholog from the largest evolutionary distance according to the ImmTree database is *C. elegans*, therefore we assume that this gene was already present in the ancestor of the whole Bilateria group, so we assign level number 7 for this gene.

Subnetworks for all ten levels were generated with the program Cytoscape [50] so that all the nodes and the corresponding edges, which emerged later than the present level, were eliminated from the interaction network. Thus, network level 0 is equivalent to the original protein-protein interaction network; whereas networks with higher level numbers represent the network at earlier steps of evolution with a fewer number of nodes and edges (Table 1). Statistics and different network parameters were calculated for the subnetworks using the igraph R library [51].

### PPI interactions for *D. melanogaster *and *C. elegans*

Experimentally derived data for low level PPI subnetworks in fruitfly and worm was acquired from the IntAct [42] and PIMRider [43] databases, respectively. Orthologs of human immunome proteins were identified in these genomes using data from the ImmTree database. Then, interactions between the immunome ortholog proteins were identified from the datasets and included to the low level networks. Interactions in the fruitfly data were introduced to the Coelomata ancestor's subnetwork (level 6) and worm data to the Bilateria ancestor's subnetwork (level 7). There were 132 immunome ortholog proteins identified in fruitfly and 27 in worm. The interaction datasets contained 13 new interactions between these proteins. These interactions were analysed similar to the human PPI network derived data. Thus, interactions were maintained in the earlier subnetworks only if both the interacting proteins were present on that level.

### Degree distribution of the subnetworks

Power law distribution was fitted to the distribution of degrees in all subnetworks. The power law exponent (α) was estimated with its standard error by maximum likelihood method.

### Average entropy of the proteins

Multiple protein sequence alignments were downloaded from the ImmTree database for each protein with an evolutionary level number higher than 0. Entropy values were calculated for each site of the alignments [47] as follows:

$$s=-\sum_{i=1}^{6}p_{i}\ln(p_{i}),$$

where *p*_*i *_is the frequency of residues from class *i *at the position. The following six classes of amino acids were used: aliphatic (A, V, L, I, M, C), aromatic (F, W, Y, H), polar (S, T, N, Q), basic (K, R), acidic (D, E) and special conformation (G, P). The arithmetic mean of the entropy was calculated for those sites, where at least 50%+1 of the sequences was present in order to avoid the overestimation of conservation caused by long unique sequence parts in the alignment, which usually appear at the ends of the alignment.

### Efficiency of the network

Global efficiency quantifies the efficiency of the network in sending information between nodes, assuming that the efficiency for sending information between two chosen nodes is proportional to the reciprocal of their distance [48]. Global efficiency was calculated as follows:

$$E=\frac{1}{N(N-1)}\sum_{i\neq j}\frac{1}{d_{ij}},$$

where *d*_*ij *_is the distance between the *i*-th and *j*-th nodes as the minimal number of edges on the shortest path between them.

### Expected efficiency of the networks

In small world networks the average path length is expected to follow *L ~ ln ln N*, where *N *is the number of nodes in the network [36]. Global efficiency is the reciprocal of the average path length [48], therefore we calculated the expected efficiency of the networks as:

$$E_{\exp}\sim \frac{1}{\ln\ln N}.$$

If we assume that the subnetworks have power law degree distribution, this model of expected efficiency can therefore be used. However, in ultra small networks the average path length is better estimated like *L ~ ln N*. Since our network models are small, a second curve for the expected efficiency was calculated as:

$$E_{\exp small}\sim \frac{1}{\ln N}.$$

### Maximum vulnerability of the networks

The vulnerability of a network was calculated using the efficiency characteristics of the networks [49]. The vulnerability, *V*_*i*_, of a network associated with the *i*-th node is

$$V_{i}=\frac{E-E_{i}}{E},$$

where *E *is the global efficiency of the network while *E*_*i *_is the global efficiency of the network without the node *i *and all of its interactions. The overall vulnerability of the network is the value of the most vulnerable node, i.e. the largest loss in performance when a node is deleted from the network. The smaller the value for vulnerability the more stable the network is against random node removal.

### Visualization of the results

Notched boxplots were used to visualize the distributions in a succinct, comparable way using the default settings for boxplots in R. For these figures, a box was plotted between the lower and upper hinges. The median of the dataset is also indicated. Whiskers were drawn toward the data extremes, up to 1.5 times the length of the box. Data points further than the whiskers are marked with circles. The notches extend to

$$\pm 1.58*\frac{Interquartile\;Range}{\sqrt{n}}$$

from the median on the sides of the boxes, and represent roughly a 95% confidence interval for the medians. If the notches of two plots do not overlap it is a strong evidence that the two medians differ.

## Competing interests

The authors declare that they have no competing interests.

## Authors' contributions

CO collected the data from the databases, implemented the perl and R scripts which perfomed the analyses and created the figures. MV designed and coordinated the project and compiled the list of immunome genes and proteins. All authors drafted the manuscript and approved its content.

## Supplementary Material

Additional file 1 Proteins of the protein interaction network of the human immune system. Basic information, including protein name, Entrez Gene ID for the coding gene, evolutionary level and degree, is listed for all the proteins in the human immunome PPI network. Click here for file

Additional file 2 Graph representation of the immunome network at the evolutionary levels. Graphs for each level are presented separately. The gene symbols are shown. Click here for file
