# Supplementary figures and images for: Efficiency of the immunome protein interaction network increases during evolution
Source: Immunome Res. 2008 Apr 22;4:4. doi: 10.1186/1745-7580-4-4 (PMC2373292; doi:10.1186/1745-7580-4-4)

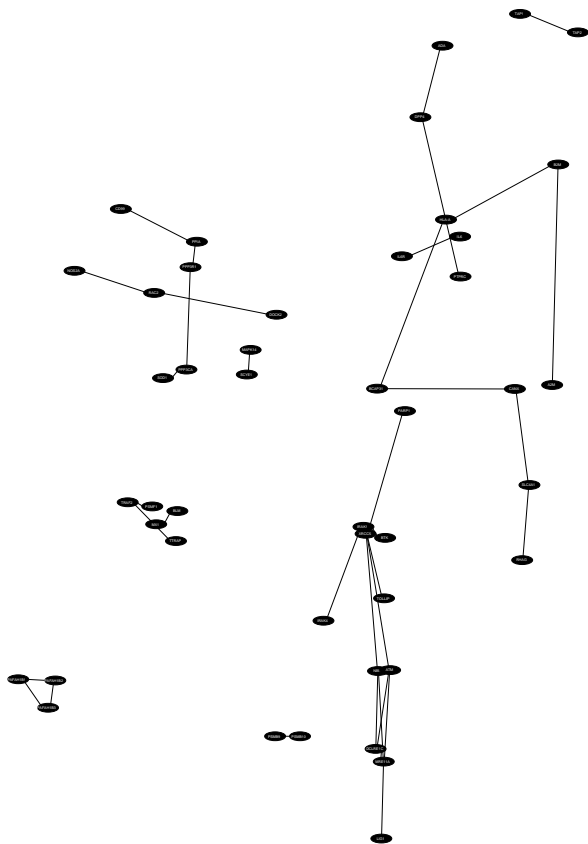

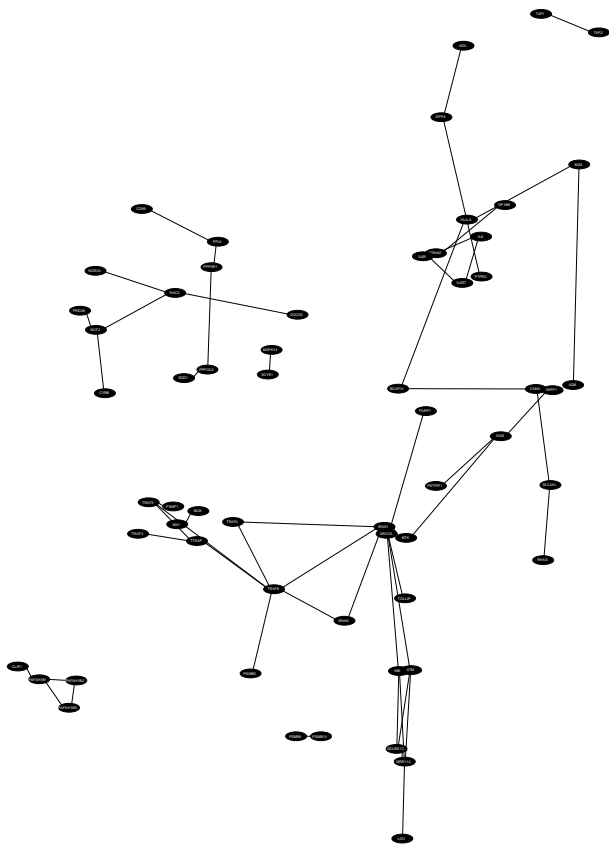

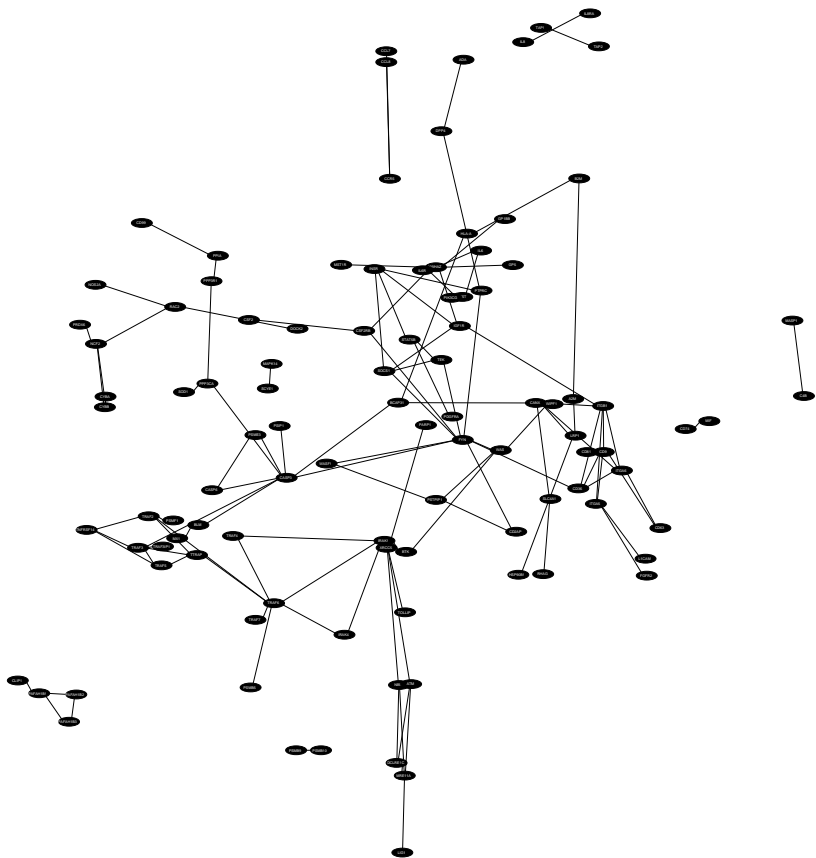

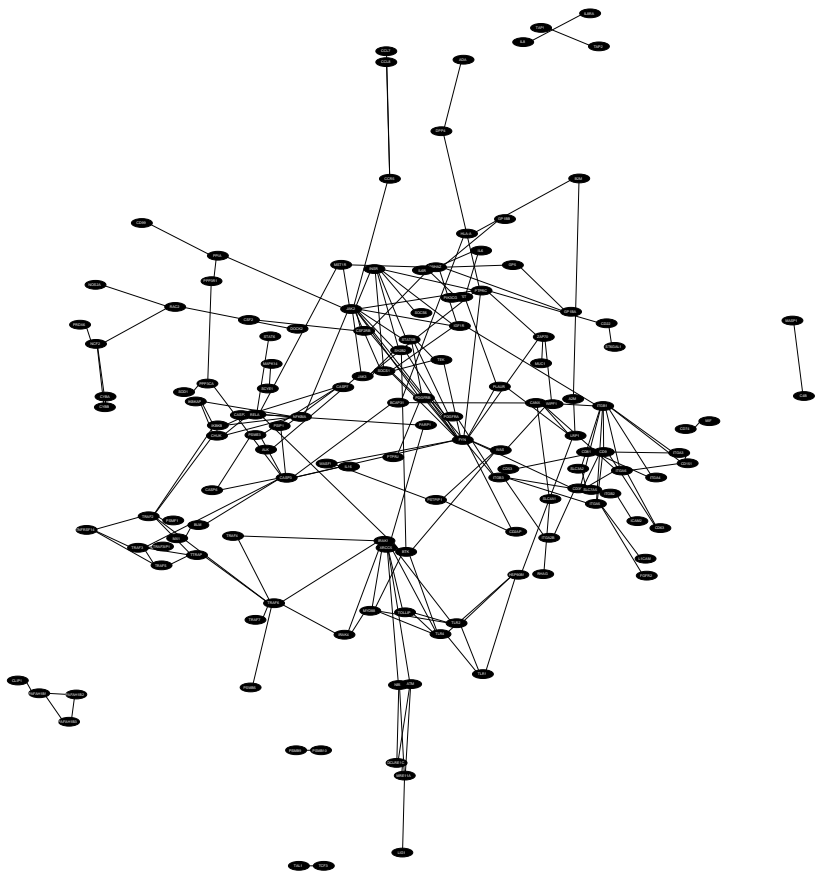

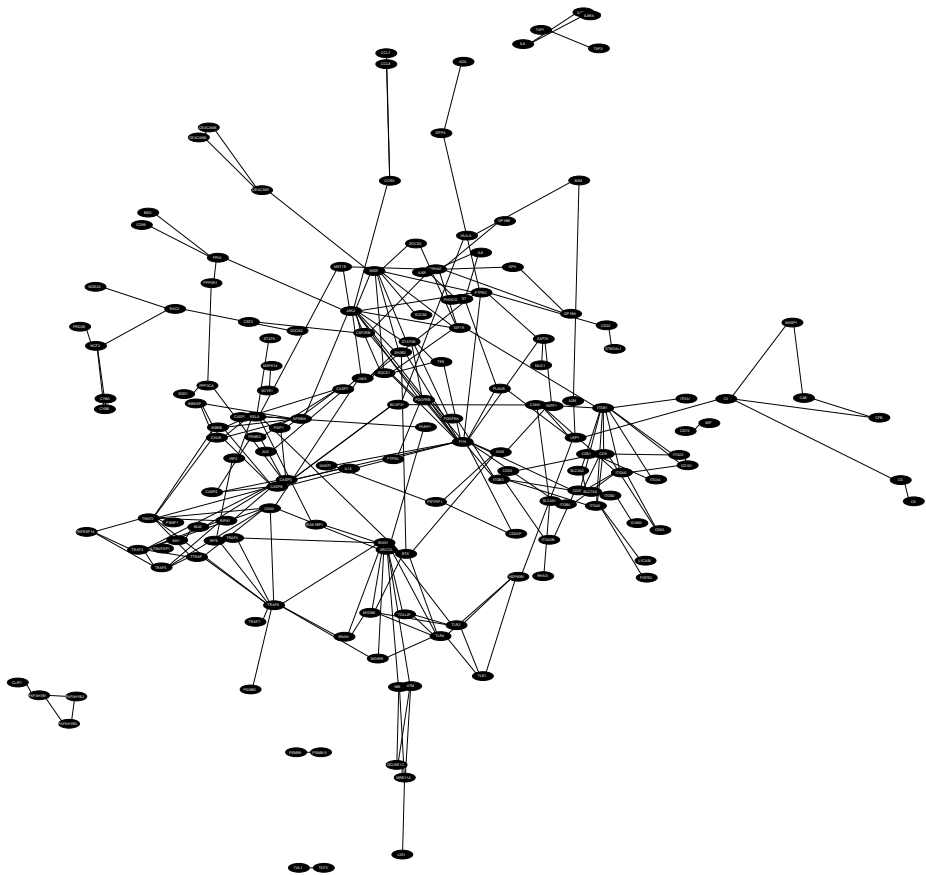

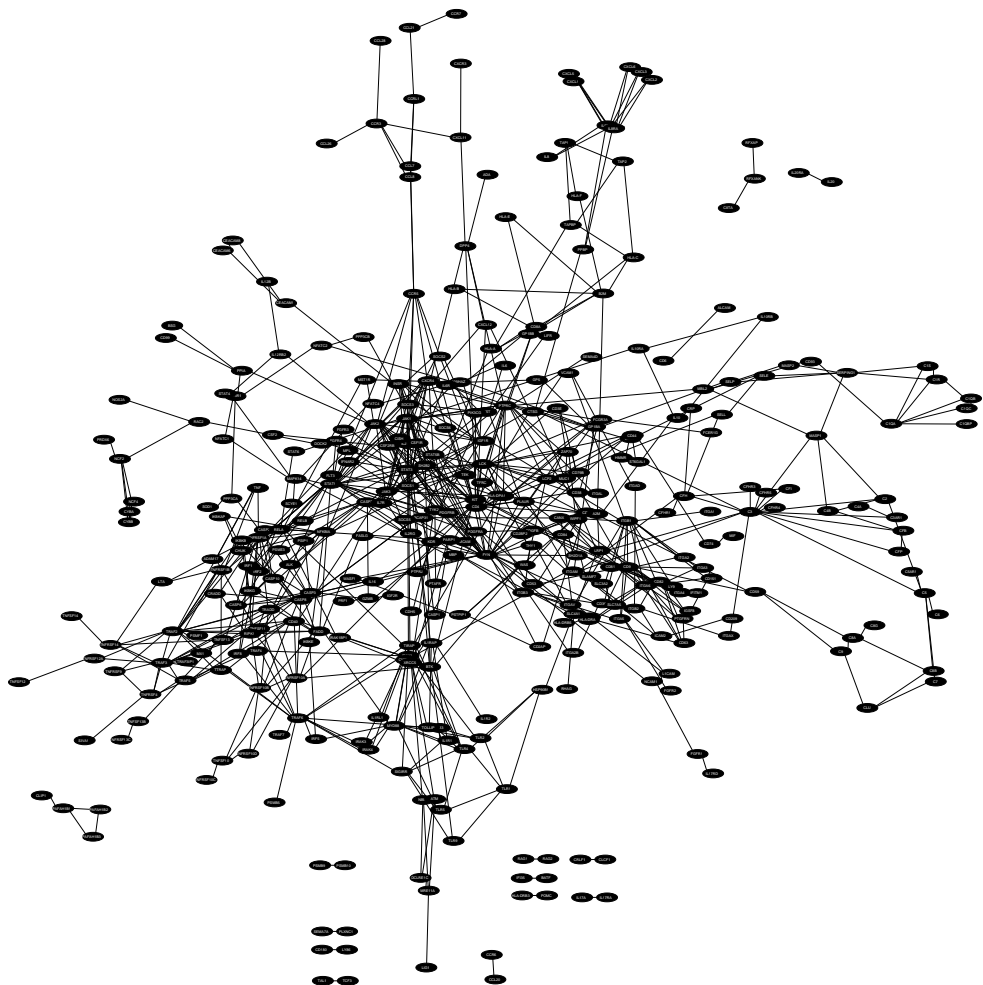

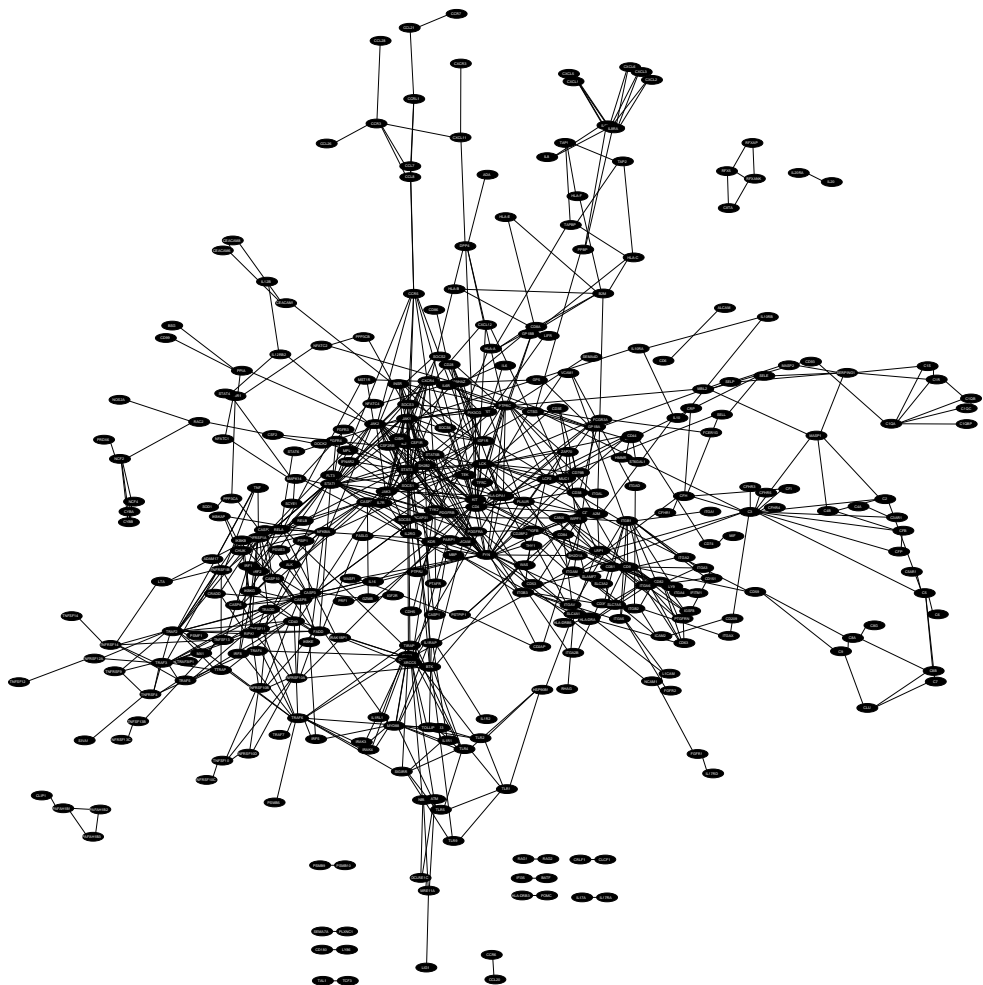

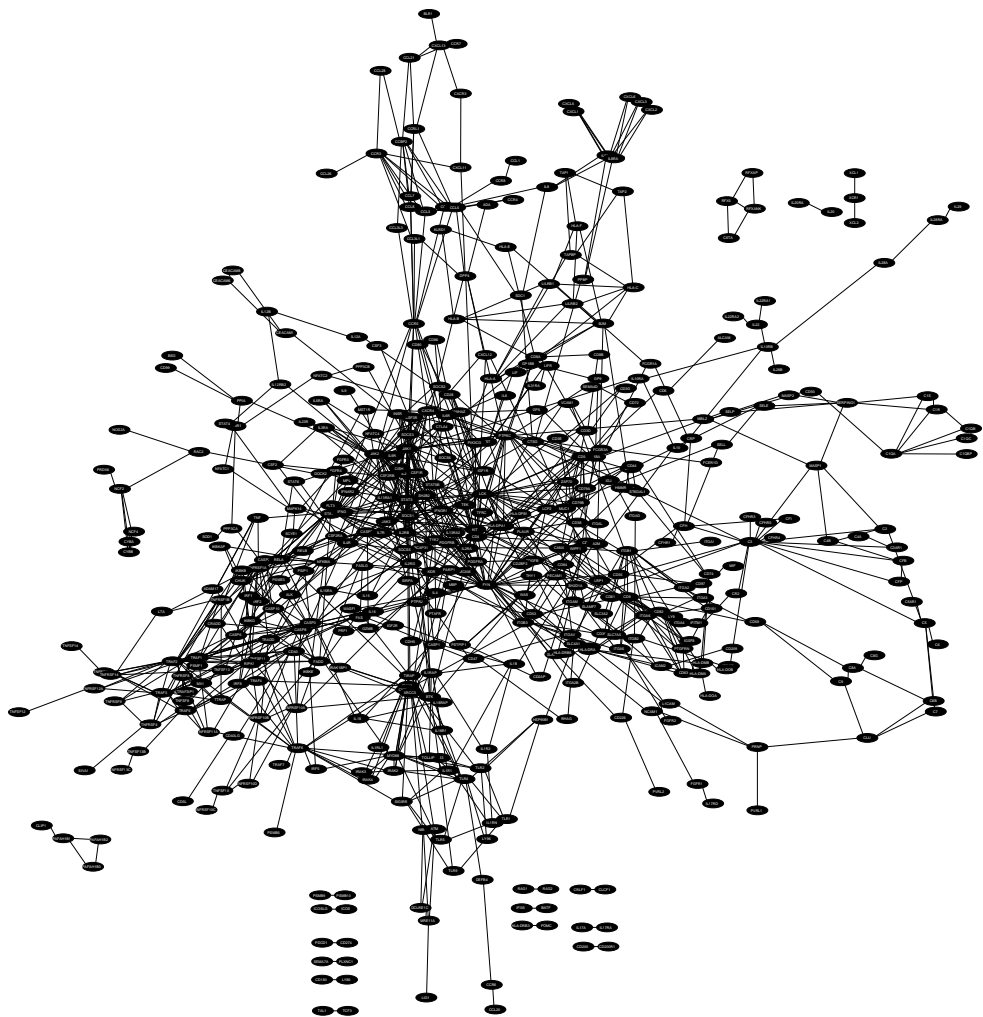

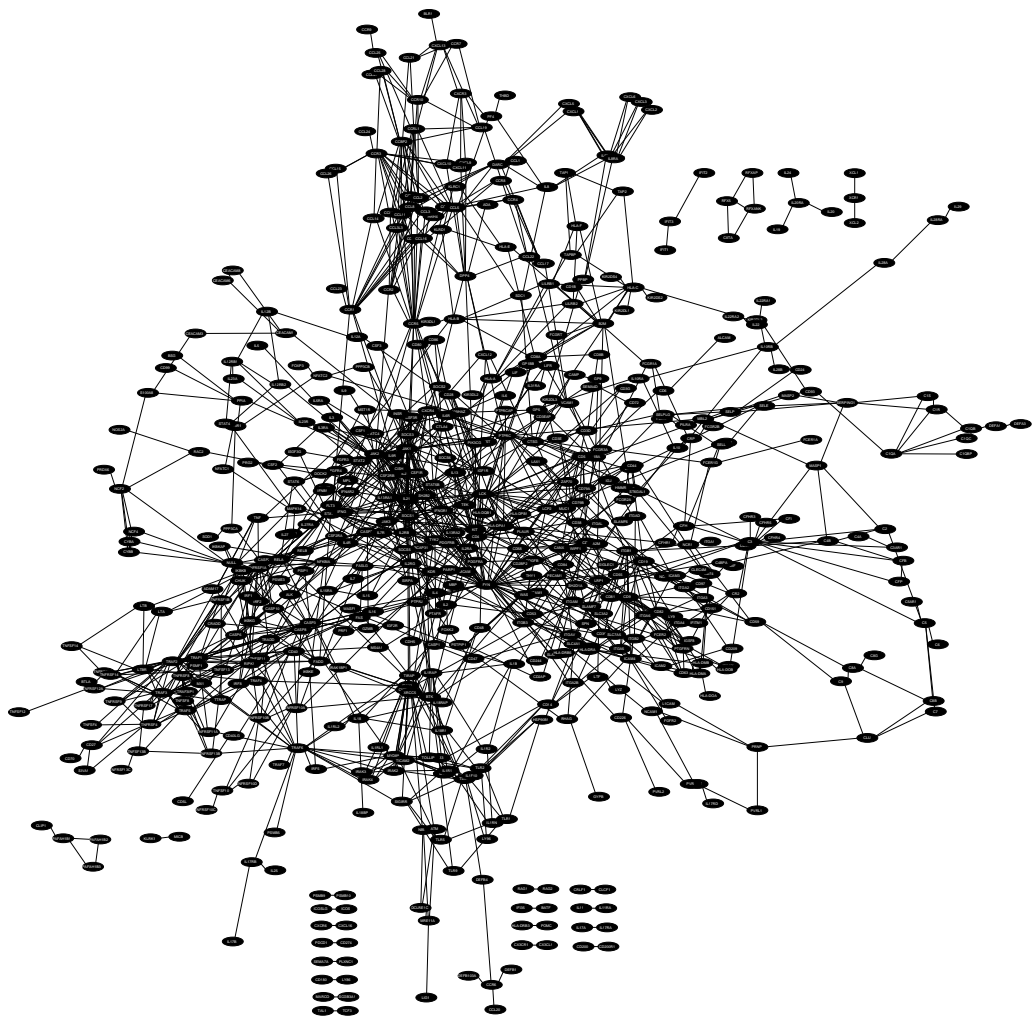

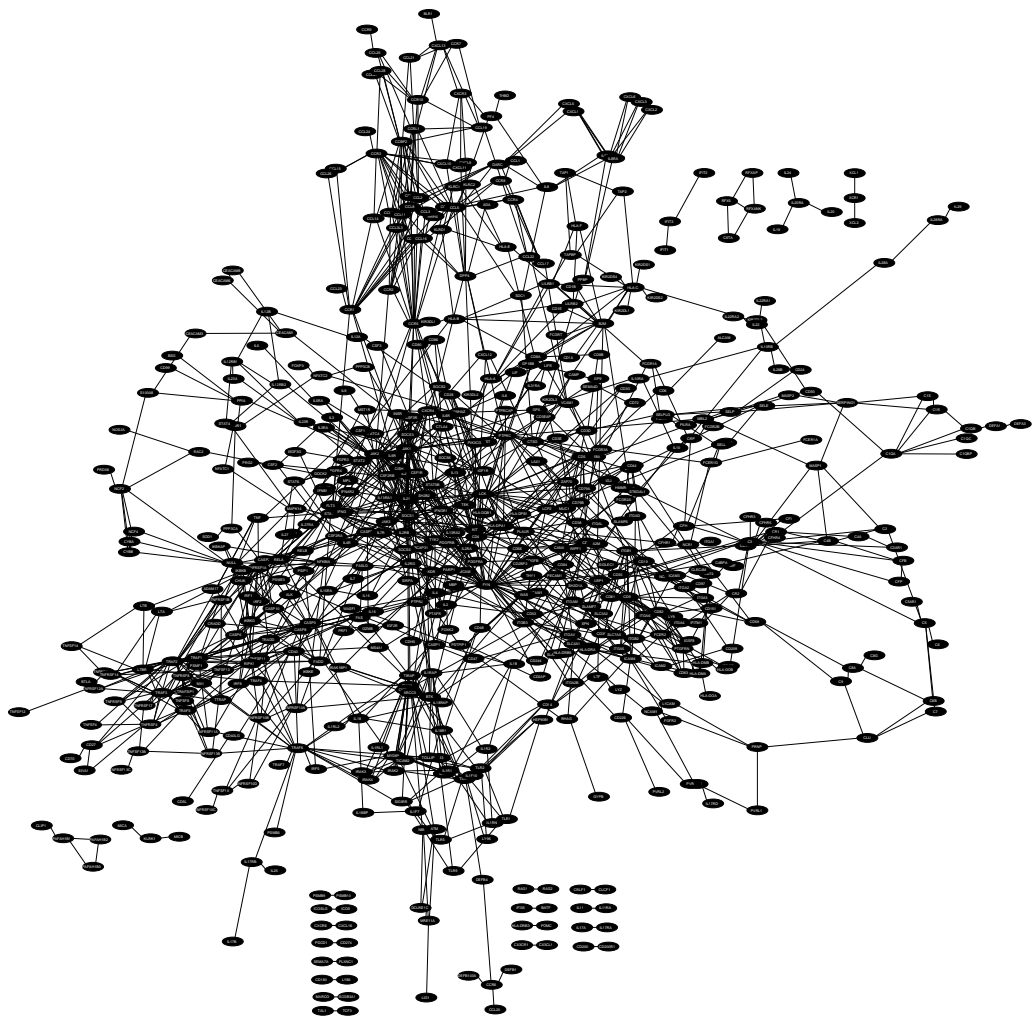

Supplement: Additional file 2 — Graph representation of the immunome network at the evolutionary levels. Graphs for each level are presented separately. The gene symbols are shown. [file 1745-7580-4-4-S2.pdf]
